# Nucleotide-dependent conformational changes direct peptide export by the transporter associated with antigen processing

**DOI:** 10.1016/j.immuni.2025.08.003

**Published:** 2025-08-29

**Authors:** James Lee, Victor Manon, Jue Chen

**Affiliations:** 1 Laboratory of Membrane Biophysics and Biology, the Rockefeller University, New York, NY 10065, USA; 2 Weill Cornell/Rockefeller/Sloan Kettering Tri-Institutional MD-PhD Program, New York, NY 10065, USA; 3 Howard Hughes Medical Institute, Chevy Chase, MD 20815, USA

## Abstract

The transporter associated with antigen processing (TAP) delivers peptide antigens from the cytoplasm into the endoplasmic reticulum (ER) for loading onto major histocompatibility complex class I (MHC-I) molecules. To examine the mechanisms of peptide transport and release into the ER, we determined cryo-electron microscopy structures of the human TAP heterodimer in multiple functional states along the transport cycle. In the inward-facing conformation, when the peptide translocation cavity within the TAP heterodimer is exposed to the cytosol, ATP binding strengthened intradomain assembly. Transition to the outward-facing conformation, when the transporter opens to the ER lumen, led to a complete reconfiguration of the peptide-binding site, facilitating peptide release. ATP hydrolysis opened the catalytically active nucleotide-binding consensus site, and the subsequent separation of the nucleotide-binding domains reset the transport cycle. These findings establish a comprehensive structural framework for understanding unilateral peptide transport, vanadate trapping, and *trans*-inhibition—an internal feedback mechanism that prevents excessive peptide accumulation and activation of the ER stress response.

## In brief

The transporter associated with antigen processing (TAP) delivers peptide antigens from the cytoplasm into the endoplasmic reticulum for loading onto MHC-I molecules. Lee et al. generate cryo-electron microscopy structures of the human TAP heterodimer in multiple states, establishing a comprehensive structural framework for understanding how ATP binding and hydrolysis drive unidirectional peptide transport.

## Graphical Abstract

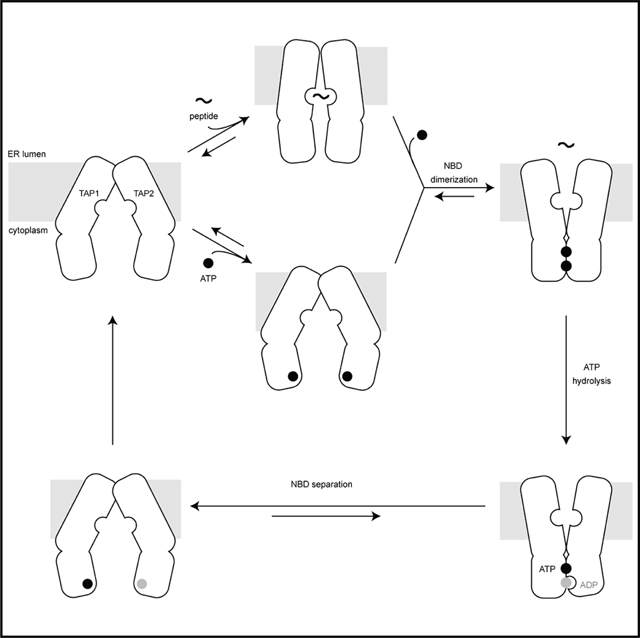

## INTRODUCTION

The transporter associated with antigen processing (TAP) is an ATP-binding cassette (ABC) transporter essential for adaptive immunity.^1,2^ TAP translocates cytosolic peptides, primarily generated by proteasomal degradation of endogenous or foreign proteins, into the lumen of the endoplasmic reticulum (ER).^3–7^ Once in the ER, these peptides are loaded onto major histocompatibility complex class I (MHC-I) molecules for presentation on the cell surface. This process enables cytotoxic T lymphocytes to detect and eliminate cells expressing non-self or aberrant peptides, such as those derived from viruses or malignancies. Mutations in TAP can result in bare lymphocyte syndrome type I (BLS-I), a rare immunodeficiency characterized by impaired antigen presentation and heightened susceptibility to infections.^8^ Additionally, numerous viruses, including herpesviruses and poxviruses, have evolved various strategies to inhibit TAP function, facilitating immune evasion and chronic infection. Structural and mechanistic understanding of TAP are foundational for investigating its function in immune surveillance and exploring potential therapeutic interventions in diseases where TAP function is compromised.

TAP is a heterodimer composed of two homologous subunits, TAP1 and TAP2.^3–7^ Each subunit consists of an N-terminal transmembrane domain (TMD0), which interacts with other ER-resident proteins, including tapasin and ERp57,^9–16^ to form the larger MHC-I peptide-loading complex.^17^ Beyond the TMD0, each subunit contains a core structure composed of six transmembrane (TM) helices that form the peptide translocation pathway and a cytosolic nucleotide-binding domain (NBD) responsible for ATP binding and hydrolysis. The core TAP heterodimer, excluding the TMD0 regions, is both necessary and sufficient for peptide translocation across the ER membrane.^18,19^ While the translocation pathway of TAP is uniquely structured to bind peptides, its NBDs share significant similarity with other ABC transporters. These domains are characterized by highly conserved sequences, including the Walker A and B motifs, which coordinate ATP; the signature motif, a hallmark of ABC transporters; and the D-loop, Q-loop, and H motif, each named after its conserved residues—aspartic acid, glutamine, and histidine, respectively.^20^

The mechanism for coupling ATP hydrolysis to substrate translocation is highly conserved in the ABC transporter family.^21^ The transport cycle of TAP is hypothesized to alternate between two primary conformational states, each exposing the peptide translocation pathway to one side of the membrane. When the NBDs are separated, the translocation pathway is open to the cytosol (inward-facing). When the NBDs form a closed dimer, the transporter opens toward the ER lumen, forming the outward-facing conformation. Upon NBD dimerization, two distinct ATPase sites are formed. One is a catalytically active consensus site composed of the Walker A and B motifs of TAP2 NBD (NBD2) and the signature motif of TAP1 NBD (NBD1). The other is a degenerate site, formed by the corresponding motifs in the opposite NBD, which binds but does not hydrolyze ATP.^22–26^

TAP adopts an inward-facing conformation in high-resolution cryo-electron microscopy (cryo-EM) structures determined in the absence of ATP.^27^ TAP recognizes and transports a wide variety of antigens—a remarkable feature of the antigen presentation pathway. Peptides, typically 8–14 residues in length,^28–30^ bind to TAP in the inward-facing state within a large transmembrane cavity formed by the TMDs. Each peptide is anchored primarily through backbone interactions at its terminal ends. By prioritizing interactions with the main-chain atoms of the peptide termini, TAP selects peptides of appropriate length while imposing minimal constraints on their sequence.^27^ This mechanism underpins TAP’s critical role in enabling the immune system to detect and respond to a broad range of antigens.

To release these peptides into the ER—some of which bind with high affinity—TAP must transition to an outward-facing state. Structural studies of the homologous transporter TmrAB,^31^ analyses of isolated NBD1,^26,32^ functional characterization of TAP variants with mutations in the NBDs,^33^ and fluorescence resonance energy transfer (FRET) measurements in permeabilized cells^34^ collectively support the concept that this transition is driven by NBD dimerization. In this study, we determined cryo-EM structures of outward-facing TAP in two distinct functional states, revealing conformational changes within the NBD dimer upon ATP hydrolysis. Additionally, we determined the structure of inward-facing TAP with either two ATP molecules or an ATP/ADP combination bound to the separated NBDs. These structures provide snapshots of TAP throughout the entire transport cycle, offering a structural model for how ATP powers unidirectional peptide transport from the cytosol to the ER.

## RESULTS

### ATP binding stabilizes the NBD1/TMD interface in the pre-translocation state

**Figure 1.**
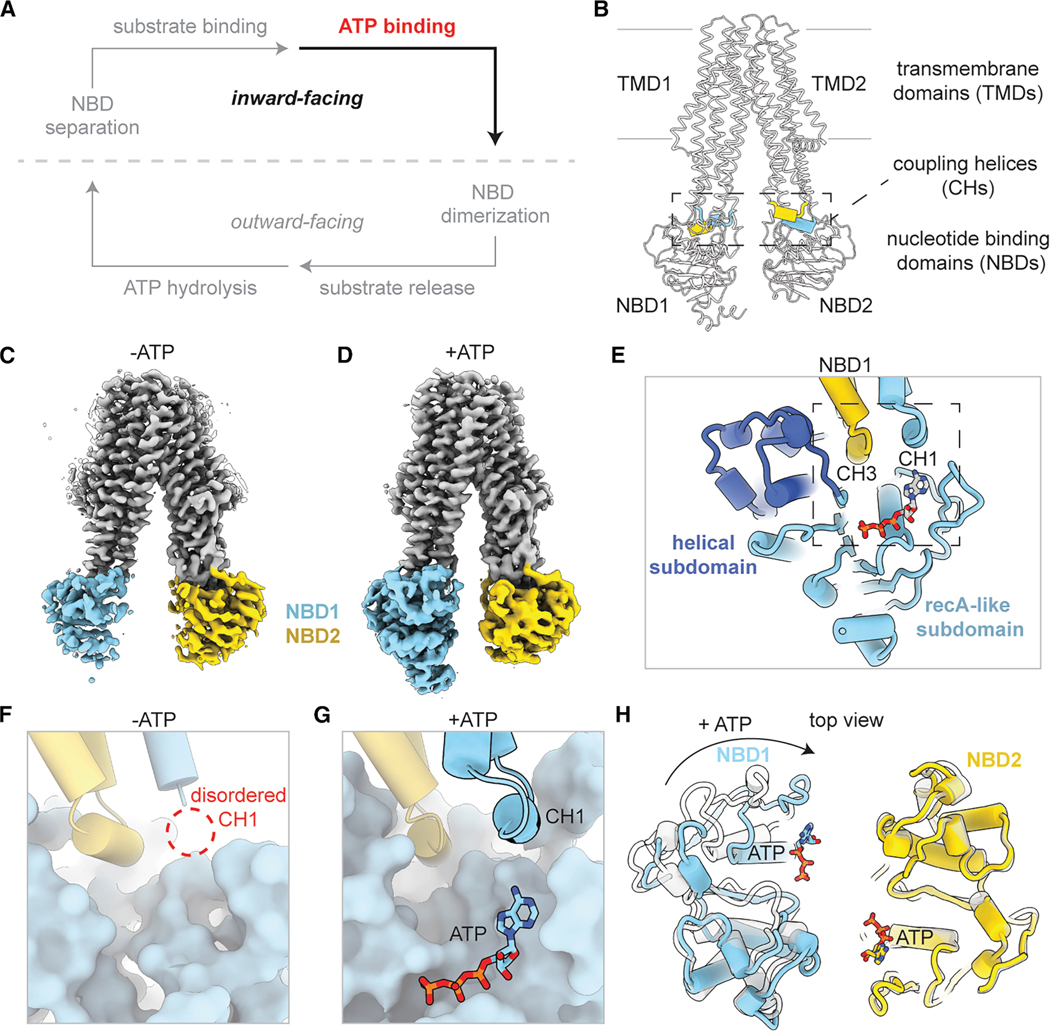
ATP binding to wild-type TAP stabilizes nucleotide-binding domain 1 (A) Schematic of the TAP transport cycle. ATP binding stabilizes the inward-facing state. (B) The coupling helices (CHs) connect the soluble nucleotide-binding domains (NBDs) to the transmembrane domains (TMDs). TAP is represented as a white ribbon. The TAP CHs are represented as colored tubes. The boundaries of the membrane are shown as silver lines. (C) Cryo-EM density of TAP bound to a 9-mer peptide has a flexible NBD1 (EMDB: EMD-41029). Densities corresponding to the TAP TMDs, NBD1, and NBD2 are colored as silver, sky blue, and gold, respectively, and contoured to 0.125 standard deviations (SDs). (D) Cryo-EM density of TAP bound to a 9-mer peptide and ATP has a rigid NBD1. Density is contoured to 0.8 SDs. (E) TAP1 CH1 and TAP2 CH3 interact with NBD1 near the ATP-binding site at the interface between TMD and NBD1. TAP is shown as cartoon tubes, and ATP is shown as sticks. (F and G) Zoomed-in view of the TMD/NBD1 interface as boxed in (E) in the absence (PDB: 8T4F) (F) or presence (G) of ATP. The dotted line represents the unresolved CH1. (H) ATP binding is associated with a rotation of NBD1 that brings the two NBDs to face each other. Superposition of TAP in the absence (white) and presence (colored) of ATP. The arrow indicates the movement of NBD1 upon ATP binding. See also Figure S1.

To analyze the effect of ATP binding, full-length wild-type (WT) TAP was incubated with 5 mM ATP and 20 μM of the high-affinity peptide RRYQKSTEL^35^ at 4° C for 15 s and immediately vitrified for cryo-EM analysis. Under this condition, TAP adopted an inward-facing conformation with the peptide bound inside the TM cavity and two ATP molecules attached to either NBD (Figures 1 and S1). We did not observe a conformation with the NBDs dimerized; thus, it is likely that we have captured TAP in an ATP-bound, pre-translocation state (Figure 1A).

The cryo-EM reconstruction was refined to an overall resolution of 3.6 Å. The densities for the core transporter, peptide, and ATP were well-resolved. However, the densities corresponding to the TMD0 regions of both TAP1 and TAP2 were absent, indicating high flexibility of these regions (Figure S1). Although the overall structure resembled the inward-facing conformation observed in the absence of ATP, local differences were apparent in NBD1 and its interface with the TMDs (Figure 1B). A common feature of cryo-EM inward-facing TAP structures in the absence of ATP^27^ was the poorly defined density for NBD1, which limited building to only a polyalanine model, even after extensive data processing (Figure 1C). By contrast, the cryo-EM map obtained in the presence of ATP revealed well-resolved density for NBD1, comparable to that of other domains (Figure 1D), allowing us to build its structure *de novo*.

The structure of the NBD and its interface with the TMDs are highly conserved within the ABC transporter family.^20,32^ Each NBD comprises two flexibly linked subdomains: a larger RecA-like subdomain and a smaller helical subdomain (Figure 1E). The NBD1/TMD interface involves two intracellular loops: coupling helix 1 (CH1) from TAP1 and CH3 from TAP2, which contact with the Q-loop and the helical subdomain in NBD1 (Figure 1E). In the absence of ATP, CH1 lacked defined density, indicating its high flexibility (Figure 1F). In the presence of ATP, CH1 adopted a well-defined structure, forming close contacts with NBD1 and the bound ATP (Figure 1G). Additionally, the linker connecting TMD1 and NBD1 (residues 485–491) also became structured in the ATP-bound state, interacting simultaneously with TMD1 and NBD1 to enhance the stability of the NBD1/TMD interface.

A comparison of the inward-facing TAP structures, with and without ATP, showed that the transmembrane domains (TMDs) and NBD2 remain almost identical (Video S1). However, ATP binding caused the RecA-like subdomain in NBD1 to rotate, moving the degenerate ATP-binding site about 8 Å closer to the molecular center (Figure 1H). As a result, the two NBDs aligned in the same “head-to-tail” configuration seen in closed dimers. γ-phosphate-induced rotation of the subdomains was observed in isolated NBD structures.^32^ Here, we demonstrate that similar conformational change occurs within the full transporter, suggesting that this rotation is an integral part of the mechanism for coupling transport to ATP hydrolysis.

Together, these results show that ATP binding to inward-facing TAP not only aligns the NBDs structurally but also stabilizes their interactions with the TMD. This process primes TAP for the transition to the NBD-dimerized, outward-facing conformation.

### The outward-facing TMDs exhibit multiple configurations

**Figure 2.**
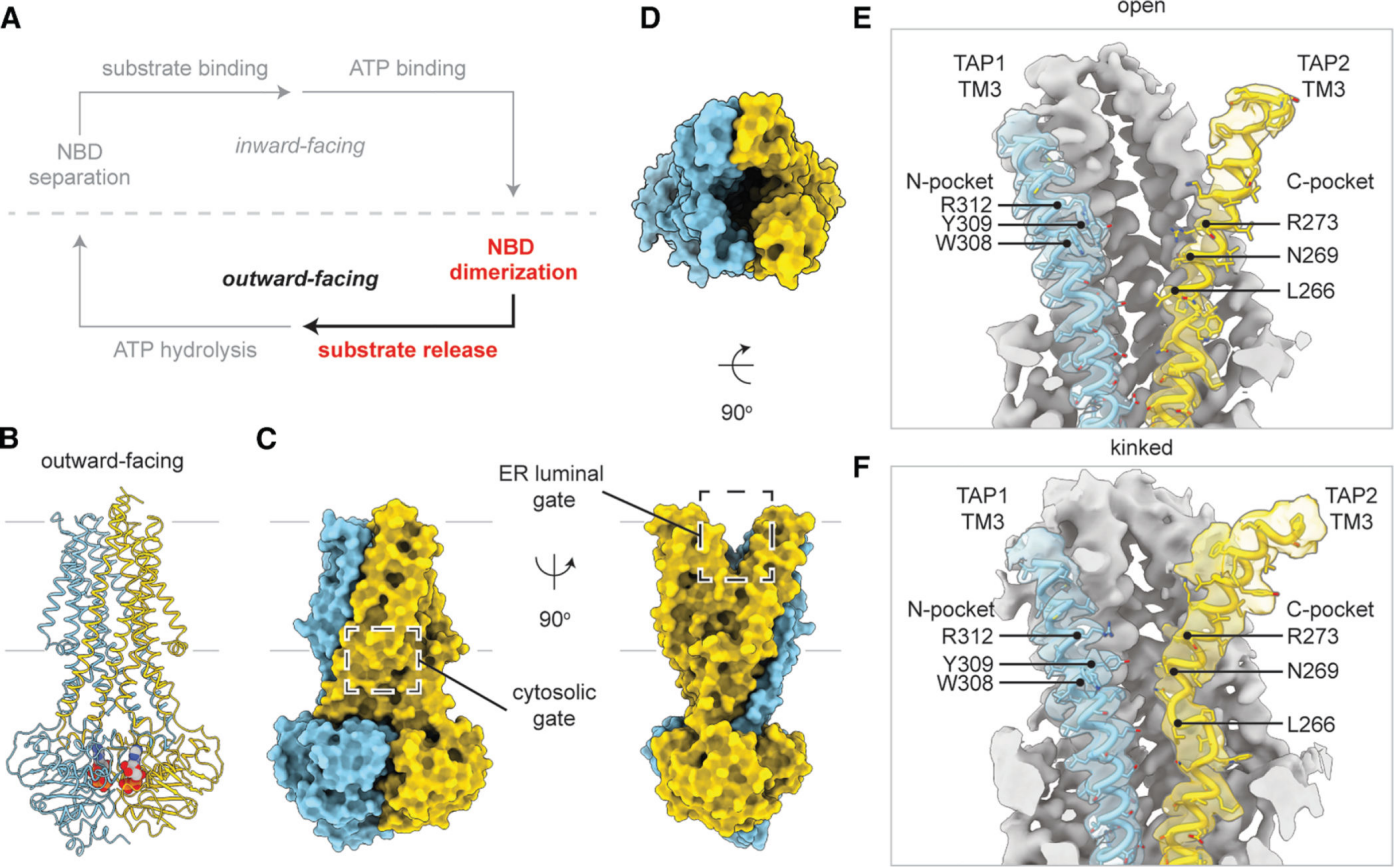
ATP binding to ATP hydrolysis-deficient TAP(EQ) adopts the outward-facing state (A) NBD dimerization stabilizes the outward-facing state. (B) Molecular model of TAP(EQ) in the NBD-dimerized outward-facing ATP-bound state. TAP1 and TAP2 are shown as ribbons and colored in sky blue and gold, respectively. ATP is shown as spheres. The boundaries of the membrane are shown as silver lines. (C) Molecular surface models of TAP(EQ) in the NBD-dimerized outward-facing ATP-bound state. The closed cytosolic and open ER luminal gates are boxed. (D) The translocation pathway is open to the ER lumen. (E and F) TAP2 TM3 is flexible and adopts two conformations in the outward-facing state: an outward-facing open (E) and kinked (F) state. The molecular models of TAP1 and TM3 are represented as cartoons with the side chains represented as sticks. Cryo-EM densities corresponding to TAP1 and TAP2 TM3 are colored in transparent sky blue and gold, respectively, and contoured to 0.12 and 0.196 SDs, respectively. The N- and C-pockets of the peptide-binding sites are labeled. See also Figures S2–S4.

To stabilize the NBDs in a dimerized conformation (Figure 2A), we replaced the catalytic glutamate in the consensus site of TAP2 with a glutamine, generating an EQ variant (TAP2 E631Q) that diminishes ATP hydrolysis while preserving ATP binding.^36^ The cryo-EM sample was prepared at 4° C in the presence of 5 mM ATP and in the absence of peptide. Unlike the WT TAP, approximately 50% of the EQ variant adopted the NBD-dimerized conformation (Figures 2B–2D and S2). The overall resolution was ∼3.1 Å. Local resolution analysis indicated that the cytosolic half of the molecule was well-defined, whereas the TMDs in the ER leaflet were more mobile (Figure S4). Subsequent focused classification revealed that TAP2 TM3 was conformationally heterogeneous: while the majority of the structures exhibited a continuous helix (Figure 2E), a subset formed a kink around P265 near the peptide-binding site (Figures 2F, S2, and S3).

The translocation pathway, as anticipated, was closed to the cytosol and open to the ER lumen (Figures 2C and 2D). Similar to the prototypical bacterial multidrug exporter Sav1866^37^ (Figure S4A), this pathway can also be accessed directly from the lipid bilayer through two lateral openings: one between TM1 and TM2 of TAP1 and another in TAP2 between TM1 and TM3 (Figure 2C). Both openings extended down to the level of the peptide-binding pockets, roughly at the membrane’s midplane (Figure S4B). Compared with the inward-facing conformation, the TM cavity in this state was narrower, and when TM3 adopted a kinked conformation, the translocation pathway became even more constricted (Figures 2E and 2F).

Although the two NBDs are functionally distinct, their structures in this conformation appeared very similar, evident by the root mean square deviation (RMSD) of 0.63 Å over 227 Cα positions (Figure S4C). It is possible that the NBD2 E631Q substitution, by eliminating the catalytic glutamate, effectively converted the consensus site into a degenerate site and thus masked subtle, local asymmetries that may be present between the two ATPase sites.

### Transition to the outward-facing state promotes substrate release

**Figure 3.**
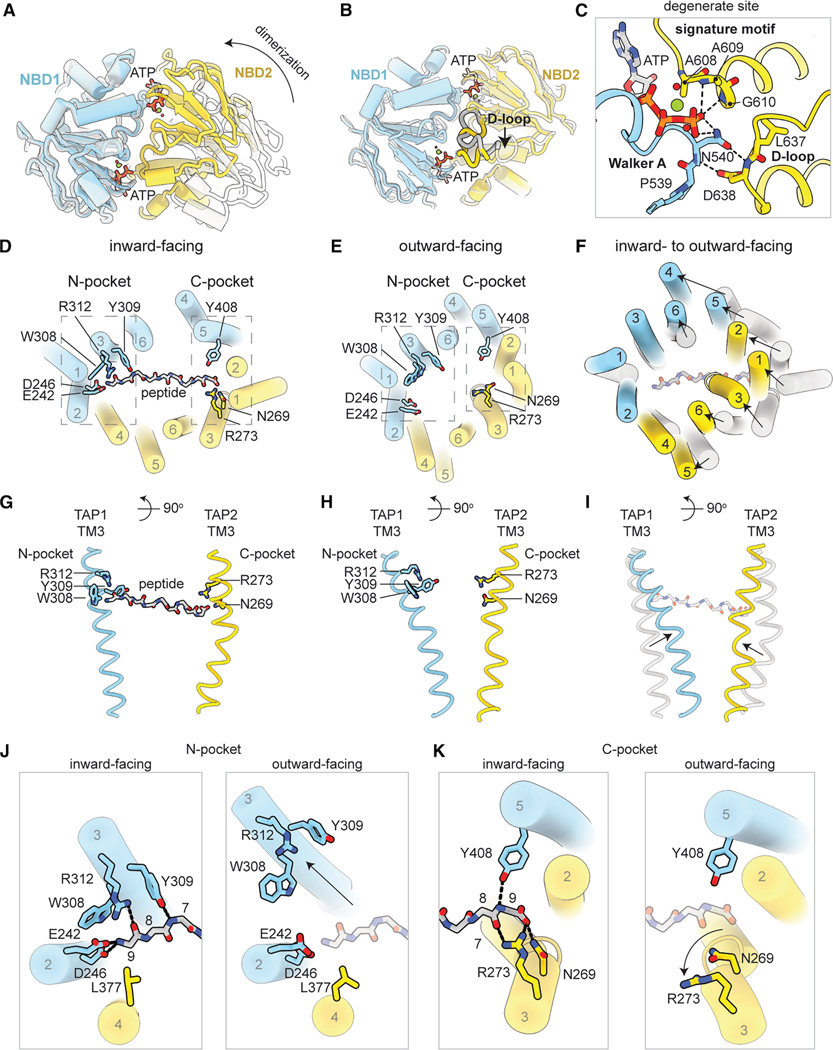
Conformational changes upon NBD dimerization in outward-facing TAP(EQ) enable substrate release (A) Global conformational changes upon NBD dimerization as viewed from the cytoplasm. Superposition of NBD1 in the NBD-separated (white) and NBD-dimerized (colored) conformations. The arrow indicates the movement of NBD2. (B) Local conformational changes in the NBDs upon NBD dimerization. Superposition of NBD1 and NBD2 individually in the NBD-separated (white) and NBD-dimerized (colored) conformations. The D-loop is highlighted, and the arrow indicates movement of the D-loop to interact with ATP in the degenerate site. (C) Zoom-in view of the degenerate nucleotide-binding site in NBD1. Hydrogen bonds are represented as dashed lines. (D and E) The TAP translocation pathway in the 9-mer peptide-bound inward-facing (D) or outward-facing (E) conformation as viewed from the ER lumen. The helices of TAP are numbered and shown as colored cartoon tubes. The main-chain backbone of the peptide substrate is shown as silver sticks. The N-pocket and C-pocket are boxed as indicated, and residues that comprise each pocket are shown as sticks. (F) Superposition of the TAP translocation pathway in the inward-facing peptide-bound (silver) and outward-facing (colored) conformations. Arrows indicate movement of the TAP helices. (G and H) Conformation of TAP TM3 in the 9-mer peptide-bound inward-facing (G) or outward-facing (H) conformation as viewed from the membrane. (I) Superposition of TAP TM3 in the inward-facing peptide-bound (silver) and outward-facing (colored) conformations. (J and K) Zoom-in of the TAP N- (J) and C- (K) pockets in the 9-mer peptide-bound inward-facing (left) or outward-facing (right) conformation. Hydrogen bonds are shown as dotted lines. The alpha-carbons of the peptide substrate are numbered. The peptide from the inward-facing state is superimposed upon the outward-facing state and is represented as transparent sticks (right). Arrows indicate movement of the TAP TM3. See also Figures S2–S4.

Transition from the ATP-bound, inward-facing to the outward-facing conformation involved global and local changes (Figure 3; Video S1). While NBD1 moved as a rigid body (Figure 3A), NBD2 underwent additional changes in the D-loop, which contained a conserved aspartate crucial for NBD dimer stabilization^33^ (Figure 3B). Upon dimerization, D638 in NBD2 shifted 3.6 Å toward the interface, forming two hydrogen bonds with N540 in the NBD1 Walker A motif to further strengthen the NBD dimer interface (Figure 3C).

NBD dimerization induced global conformational changes that reorient the translocation pathway, closing its cytosolic route and opening it widely toward the ER lumen (Figures 3D–3I; Video S1). These changes were accompanied by a complete reconfiguration of the peptide-binding site. In the NBD-separated conformation, TAP binds a peptide through two distal pockets, each interacting with opposite ends of the peptide^27^ (Figures 3D and 3G). The N-pocket, primarily composed of charged and aromatic residues in TM2 and TM3 of TAP1, forms extensive contacts with the first three residues of the peptide. Meanwhile, the C-pocket, involving residues mainly in TM 2 and TM3 of TAP2, secures the peptide’s last residue and C terminus within the translocation pathway. In the NBD-dimerized conformation, the distance between the N- and C-pockets was reduced by approximately 10 Å (Figures 3E and 3H), rendering the binding site incompatible with a 9-mer peptide in its extended conformation (Figures 3F and 3I). In addition, local structural rearrangements within each binding pocket further reduced peptide affinity. In the N-pocket, TM3 of TAP1 shifted away, disrupting two hydrogen bonds with the peptide (Figure 3J). In the C-pocket, TM3 of TAP2 rotated, moving R273 out of hydrogen-bonding range (Figure 3K).

These structural changes not only altered the access of the peptide-binding site but also reorganized the binding pockets to reduce their affinity for peptides. In this conformation, the peptide would be released into the ER by simple diffusion. Additionally, the observed flexibility of TAP2 TM3 may also play a role: by forming the kinked conformation, the C-pocket is nearly demolished, and the translocation pathway becomes so constricted that peptide rebinding is highly unlikely (Figure 2F).

### Asymmetric opening of the NBDs upon ATP hydrolysis

**Figure 4.**
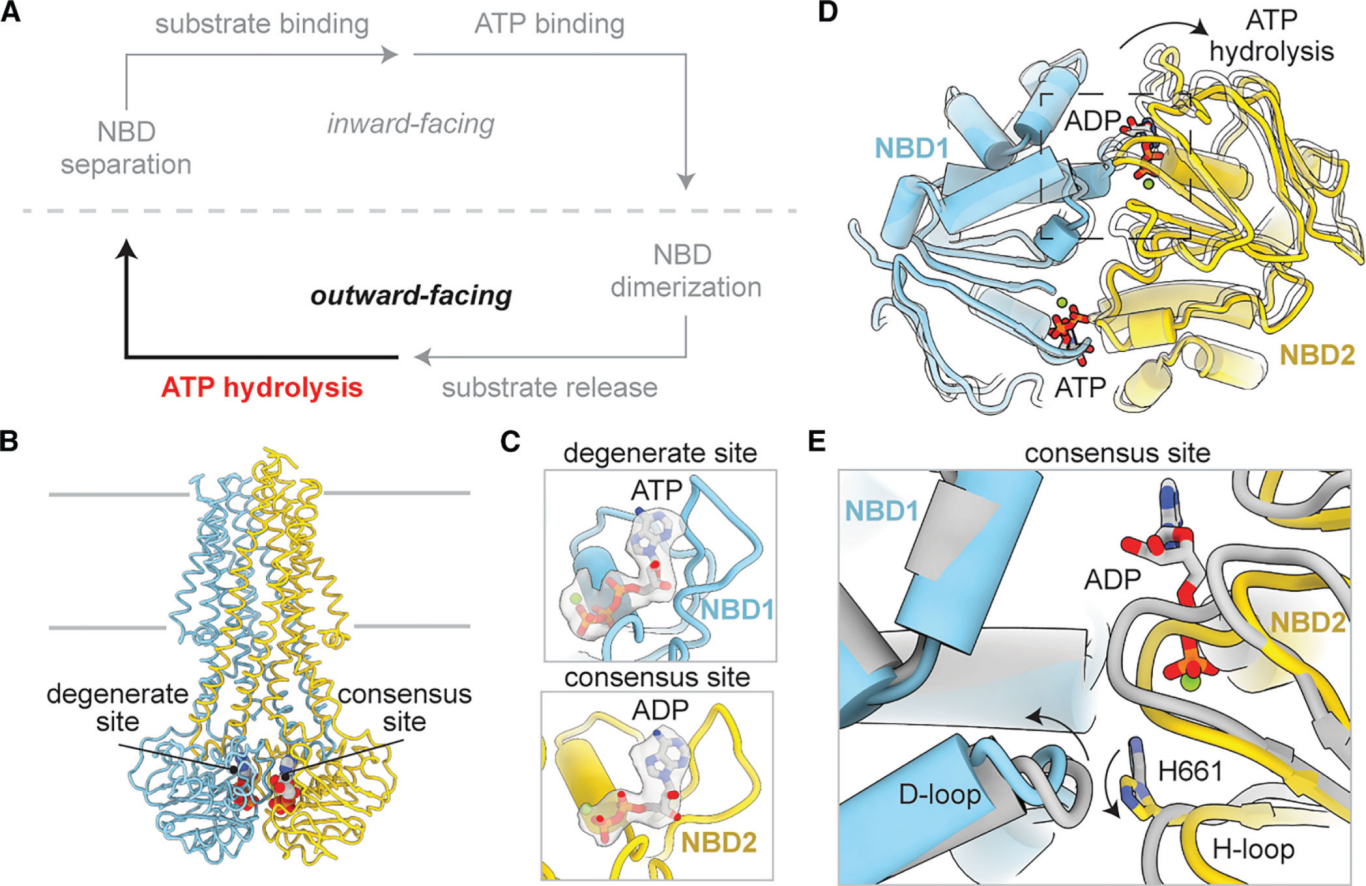
WT TAP incubated with ATP at 37° C captures a post-hydrolytic outward-facing state with asymmetrically separated NBDs (A) The post-hydrolytic state can adopt an outward-facing state. (B) Molecular model of WT TAP in the NBD-dimerized outward-facing post-hydrolytic state. The degenerate and consensus ATP-binding sites are labeled. (C) ATP is bound in the NBD1 degenerate site, and ADP is bound in the NBD2 consensus site. ATP is shown as sticks, and the magnesium atom is shown as spheres. Density corresponding to bound nucleotide is shown as a gray surface and contoured to 0.22 SDs. (D) Superposition of NBD1 before (white) and after (colored) ATP hydrolysis as viewed from the cytoplasm. Arrows indicate conformational changes in NBD2 after ATP hydrolysis. (E) Zoom-in view of the NBD2 consensus site as boxed in (D). Arrows indicate local conformational changes in the D-loop of NBD1 and the H-loop of NBD2 after ATP hydrolysis. See also Figure S5.

Since the NBD-dimerized TAP(EQ) structure likely represents the pre-hydrolysis state, where ATP is positioned for hydrolysis and the peptide has already been released to the ER, we aimed to capture the post-hydrolysis state of TAP (Figure 4A). To achieve this, WT TAP was incubated with 10 mM ATP at 37° C for 1 min to enable ATP hydrolysis, and the sample was subsequently vitrified for cryo-EM analysis. Under these active turnover conditions, both NBD-separated and dimerized conformations were observed in approximately equal abundance (Figure S5).

The NBD-dimerized reconstruction was refined to 3.2 Å resolution (Figures 4B and S6), showing clear density for ATP at the degenerate site and ADP/Mg^2+^ in the consensus site (Figure 4C), suggesting it represented a post-hydrolytic state before NBD separation. The overall structure closely resembled the pre-hydrolytic, outward-facing conformation (Figure 3), except for a subtle opening at the consensus site (Figure 4D; Video S1). Following ATP hydrolysis, the H-loop of NBD2 (which contains the conserved His residue coordinating the gamma phosphate^38^) and the D-loop of NBD1 shifted away, resulting in an opening of the dimer interface by approximately ∼3 Å (Figure 4E). No density corresponding to inorganic phosphate (Pi) was observed, suggesting it had already diffused out of the binding site prior to opening of the NBD dimer interface. The asymmetric opening is consistent with the functional asymmetry of the two ATPase sites, as ATP hydrolysis only occurs at the consensus site.

**Figure 5.**
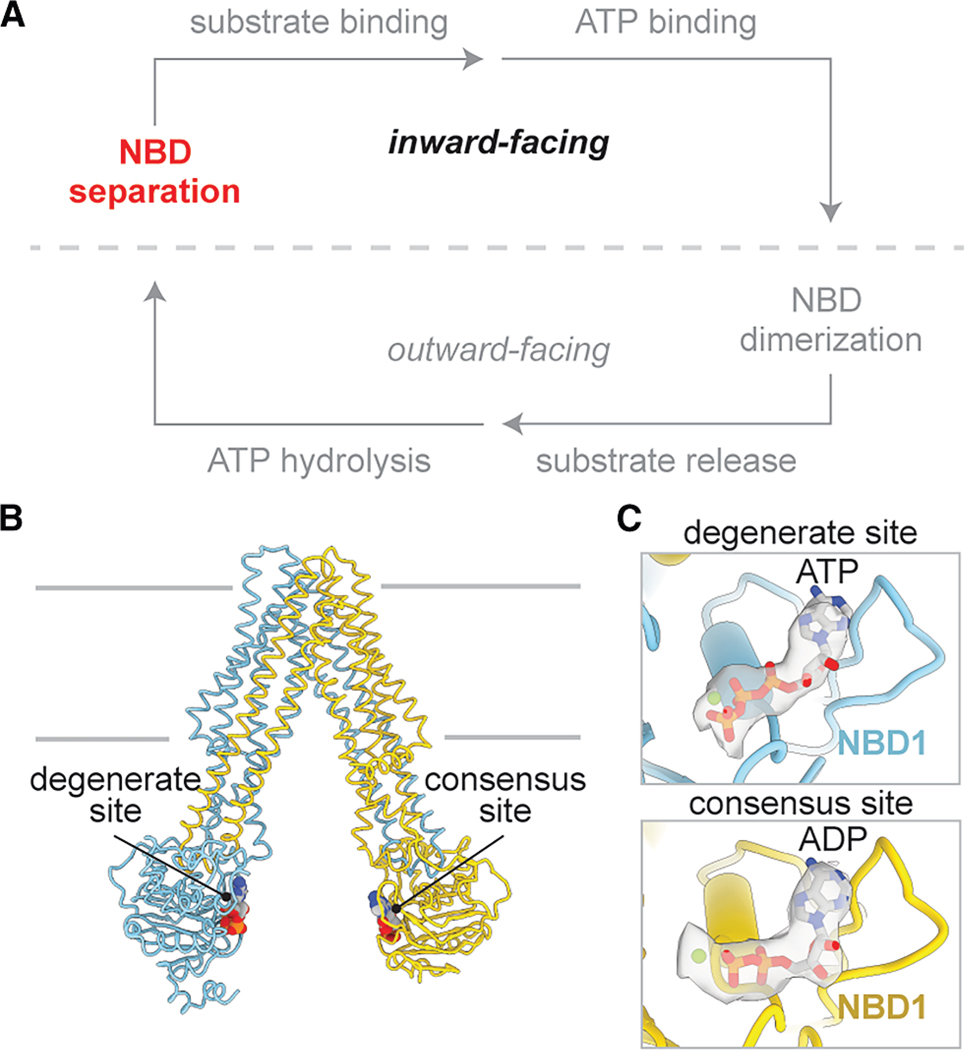
WT TAP incubated with ATP at 37° C also adopts the NBD-separated post-hydrolytic conformation (A) NBD separation resets the transport cycle. (B) Molecular model of WT TAP in the NBD-separated outward-facing post-hydrolytic state. Bound nucleotide is shown as sticks, and the magnesium atom is shown as spheres. The degenerate and consensus ATP-binding sites are labeled. (C) ATP is bound in the NBD1 degenerate site (top), while ADP is bound in the NBD2 consensus site (bottom). Density corresponding to bound nucleotide is shown as a gray surface and contoured to 0.33 SDs. See also Figure S5.

The fully separated NBD structure was resolved to a resolution of 3.7 Å (Figures 5A, 5B, and S5), revealing that ATP remained bound at the degenerate site in NBD1 (Figure 5C). The density at the consensus site in NBD2 was weaker and aligned more closely with ADP (Figure 5C). The TMDs have fully reverted to the inward-facing configuration, ready to accept substrate (Figure 5A). We interpret this structure as representing a post-hydrolysis state, where the TMDs are poised to recruit a new peptide, and the NBDs are separated to facilitate nucleotide exchange.

## DISCUSSION

This study, in conjunction with a recent publication,^27^ has resolved the structure of TAP in six distinct conformational states, allowing us to piece together the complete peptide transport cycle. In the absence of a ligand, TAP adopts an inward-facing conformation with the NBDs widely spaced. Peptide and ATP bind independently,^39^ each inducing distinct conformational changes. A peptide of the appropriate length engages both binding pockets within the transmembrane cavity, drawing the TMDs and NBDs closer together. ATP binding stabilizes and strengthens the NBD1/TMD interactions. At a physiological temperature of 37° C, these coordinated changes drive isomerization to the outward-facing state, where NBD dimerization opens the transmembrane cavity to the ER lumen and reconfigures the binding pockets, reducing their affinity for peptide release. ATP hydrolysis in the consensus site disrupts the NBD interface, ultimately resulting in complete NBD separation. Releasing ADP in exchange for ATP restarts a new cycle. This mechanism, in which NBD dimerization drives substrate release prior to ATP hydrolysis, is analogous to the drug transporters Pgp,^40^ ABCG2,^41^ MRP1,^42, 43^ and MRP2,^44, 45^ highlighting a conserved principle within the ABC transporter family.

Consistent with FRET studies,^34^ we observe that the transition to the outward-facing conformation is highly temperature-dependent. At 4° C, WT TAP predominantly remains in the inward-facing conformation, even in the presence of peptide and ATP. The outward-facing structure was captured either by incubating TAP with ATP at 37° C or by introducing the E631Q substitution to prevent ATP hydrolysis. One possible explanation for the temperature dependence is that the higher thermal energy at 37° C compared with 4° C is required to overcome the energy barrier between the inward- and outward-facing conformations. In this scenario, the increased thermal energy and molecular motion allow the NBDs to dimerize with sufficient frequency to be observed by cryo-EM.

For decades, vanadate has been widely used to trap ABC transporters in a conformation that presumably mimics the transition state of ATP hydrolysis.^46–49^ In the structure of the maltose transporter trapped with vanadate, ADP is tightly bound, with vanadate occupying the position of the gamma-phosphate group of ATP.^50^ Vanadate likely binds to an intermediate corresponding to the NBD-cracked-open structure observed in TAP, occurring after Pi has been released but before ADP is discharged and the NBDs fully dissociate (Figure 4). In this conformation, vanadate can access the consensus ATPase site to mimic Pi and stabilize the NBD dimer.

Finally, the structure of the outward-facing TAP also provides a structural basis for understanding *trans*-inhibition, in which peptide transport halts when the luminal peptide concentration reaches 16 μM.^33,51^ In this conformation, although both the N- and C-binding pockets are restructured, they likely retain the ability to bind peptides with lower affinity. At micromolar concentrations, peptides may occupy the outward-facing cavity by attaching to one of these pockets or by adopting a bent or different conformation, thereby preventing TAP from actively transporting additional peptides into the ER lumen.

Together, these structural insights reveal how TAP harnesses ATP binding and hydrolysis to drive unidirectional peptide translocation across the ER membrane. By capturing multiple conformational states throughout the transport cycle, we provide a molecular framework for understanding how binding pocket remodeling and domain dynamics are coordinated to enable efficient substrate release. This work not only advances our mechanistic understanding of antigen processing but also establishes a foundation for future studies on immune modulation and viral immune evasion strategies targeting TAP.

### Limitations of the study

The reported cryo-EM structures were prepared using detergent-solubilized full-length TAP. Although detergents are widely used for purifying membrane proteins, they are not identical to the cellular membrane environment. Full-length TAP contains two N-terminal TMD0 domains in addition to the core transporter. Only the core transporter is necessary for TAP peptide transport function.^18, 19^ The TMD0 domains are responsible for interacting with the larger peptide loading complex (PLC) to connect TAP to MHC-I.^17,52^ Density in our reconstructions corresponding to the canonical core TAP transporter was well resolved, but density for the TMD0 domains was incomplete. Future studies of TAP together with the PLC and in a cellular membrane environment will explore how the conformational changes in TAP during peptide transport influence peptide loading onto MHC-I.

## RESOURCE AVAILABILITY

### Lead contact

Requests for further information and resources should be directed to and will be fulfilled by the lead contact, Jue Chen (juechen@rockefeller.edu).

### Materials availability

All plasmids and reagents generated in this study are available from the lead contact.

### Data and code availability

Cryo-EM densities have been deposited in the Electron Microscopy Data Bank (EMDB) under the accession codes EMDB: EMD-49045, EMD-49046, EMD-49047, EMD-49048, EMD-49049, and EMD-49050. The corresponding atomic models have been deposited in the Protein Data Bank (PDB) under the accession codes PDB: 9N61, 9N62, 9N63, 9N64, 9N65, and 9N66. The raw movies have been deposited in the Electron Microscopy Public Image Archive (EMPIAR) under the accession codes EMPIAR: 12685, 12686, and 12687. Any additional data reported in this paper are available from the lead contact upon request.

## STAR★METHODS

### EXPERIMENTAL MODEL AND STUDY PARTICIPANT DETAILS

#### Cell culture

*Spodoptera frugiperda* Sf9 cells (ATCC CRL-1711) were cultured in Sf-900 II SFM medium (Gibco) supplemented with 5% (v/v) heat-inactivated fetal bovine serum (FBS) (Gibco) and 1% (v/v) antibiotic-antimycotic (Gibco) at 27°C. HEK293S GnTI- cells (ATCC CRL-3022) were cultured in Freestyle 293 medium (GIBCO) supplemented with 2% (v/v) FBS at 37°C with 8% CO _2_ and 80% humidity. TAP KO cells were generated as previously described. All commercial cell lines were authenticated by their respective suppliers. Commercial cell lines were tested monthly for mycoplasma contamination by PCR using a Universal Mycoplasma Detection Kit (ATCC) and verified to be negative.

### METHOD DETAILS

#### Protein expression

Human TAP constructs were expressed as previously described.^27^ Bacmids encoding human TAP fused to a PreScission Protease-cleavable GFP tag were generated in *Escherichia coli* DH10Bac cells (Invitrogen). Baculoviruses were harvested from Sf9 cell media by filtering through a 0.22 μm filter and amplified three times before using for cell transduction. Proteins were expressed in 2L of HEK293S GnTI- cells infected with 5% (v/v) of baculovirus at a density of 2.5–3.0 × 10^6^ cells/ml. Cells were induced with 10 mM sodium butyrate 8–12 hours after infection and cultured at 30°C for another 48 hours. Cells were harvested, snap frozen in liquid nitrogen, and stored at −80° C.

#### Protein purification

Human TAP constructs were purified as previously described. Cells were thawed and resuspended in lysis buffer containing 50 mM HEPES (pH 6.5 with KOH), 400 mM KCl, 2 mM MgCl_2_, 1mM dithiothreitol (DTT), 20% (v/v) glycerol, 1 μg ml^−1^ pepstatin A, 1 μg ml^−1^ leupeptin, 1 μg ml^−1^ aprotinin, 100 μg ml^−1^ soy trypsin inhibitor, 1 mM benzamidine, 1 mM phenylmethylsulfonyl fluoride (PMSF) and 3 μg ml^−1^ DNase I. For samples used for structural analysis, all buffers were supplemented with 1 mM ATP starting from cell lysis. Cells were lysed by three passes through a high-pressure homogenizer at 15,000 psi (Emulsiflex-C3; Avestin). Unbroken cells and cell debris were removed by one low speed spin at 4000g for 15 min at 4°C. The supernatant was subjected to a second round of ultracentrifugation at 100,000 x g for 1 hour at 4°C in a Type 45Ti rotor (Beckman) to pellet cell membranes. Membranes were resuspended by manual homogenization in a dounce in lysis buffer supplemented with 1% glycol-diosgenin (GDN) (Anatrace) and incubated for 1 hour at 4°C. The insoluble fraction was removed by centrifugation at 75,000g for 30 min at 4°C and the supernatant was applied to NHS-activated Sepharose 4 Fast Flow resin (GE Healthcare) conjugated with GFP nanobody pre-equilibrated in lysis buffer. After 1 hour, the resin was washed with 10 column volumes of wash buffer containing 50 mM HEPES (pH 6.5 with KOH), 400 mM KCl, 10% glycerol, 1 mM DTT, and 0.01% GDN. To cleave off the GFP tag, PreScission Protease was added to a final concentration of 0.35 mg ml^−1^ and incubated for 12 hours at 4°C. The cleaved protein was eluted with 5 column volumes of wash buffer and collected by passing through a Glutathione Sepharose 4B resin (Cytiva) to remove the PreScission Protease. The eluate was then concentrated using a 15 ml Amicon spin concentrator with a 100-kDa molecular weight cutoff membrane (Millipore) and purified by size exclusion chromatography (SEC) using a Superose 6 Increase 10/300 column (GE Healthcare) pre-equilibrated with SEC buffer containing 50 mM HEPES (pH 6.5 with KOH), 200 mM KCl, 1 mM DTT and 0.004% GDN. Peak fractions were pooled using a 4 ml Amicon spin concentrator with a 100-kDa molecular weight cutoff membrane (Millipore) and used immediately for grid preparation or hydrolysis measurements.

#### Cryo-EM grid preparation and data acquisition

TAP purified from gel filtration was concentrated to ∼5–6 mg ml ^−1^ and, where appropriate, incubated with 150 μM of the corresponding peptide on ice for 30 min. An additional 10 mM of ATP was applied to each sample where appropriate before freezing. To capture the post-hydrolytic state, samples were incubated at 37°C for 1 min before freezing. Grids were prepared by applying 3–3.5 μL of sample onto a glow discharged Quantifoil R0.6/1.0 400 mesh holey carbon Au grid with no wait time. The grids were blotted for 3 sec with a blot force of 20 and plunged frozen into liquid ethane using an FEI Mark IV Vitrobot at 6°C and 100% humidity.

All cryo-EM data were collected using a 300 kV Titan Krios transmission electron microscope equipped with a Gatan K3 Summit direct electron detector. All micrographs were collected using SerialEM^53^ in super-resolution mode. Data collection parameters for each sample are summarized in Table S1.

#### Image Processing

Image processing workflows are summarized in Figures S1, S2, S5; Table S1. Super-resolution image stacks were gain-normalized, binned by 2, and motion corrected using MotionCor2.^54^ Contrast transfer function parameters were estimated using CTFFIND4.^55^ Particle picking for all datasets were initially carried out with crYOLO^64^ using its general model, extracted in RELION, ^57^ and imported into cryoSPARC.^58^ The picked particles were subjected to multiple rounds of 2D classification, and the resulting particles were subjected to *ab initio* reconstruction with a maximum resolution set to 9Å. Non-uniform refinement of the best class with the most complete density for the NBDs resulted in a medium-resolution reconstruction of TAP with well-resolved transmembrane helices and NBD2, but with an invisible NBD1. To improve the density of NBD1 in the inward-facing state, all the particles from 2D classification were subjected to iterative rounds of heterogenous refinement using the best reconstruction and a decoy reconstruction with a disordered NBD1 as input models. The resulting particles that gave reconstructions with the most complete NBD1 were then subjected to tandem non-uniform refinement followed by local refinement with a protein mask excluding the micelle.

For TAP(EQ) with ATP dataset, the best particles from these reconstructions were used to train separate Topaz models for both the inward-facing and outward-facing conformations that was used to repick all the micrographs. Particles from both models were combined, duplicates were removed, and subject to the iterative 2D and 3D classification workflow described above. For TAP(EQ) in the outward-facing state, a consensus particle stack was generated consisting of 287,819 particles. This particle stack was imported into Relion using the csparc2star.py script^62^ and subject to Bayesian particle polishing,^62^ and refined again in cryoSPARC. Bayesian particle polishing did not improve the other cryo-EM maps.

For TAP(EQ) and ATP in the outward-facing kinked state, the consensus 287,819 particle stack for the outward-facing state was subject to extensive 3D classification in Relion without alignment using a mask excluding the micelle and limiting the resolution E-step to 6Å. Two classes exhibited a difference in the conformation of TAP2 TM3 and were subject to another two rounds of 3D classification in Relion without alignment without limiting the resolution E-step. In both rounds, one class exhibited more continuous density in the TM helices. The final particle stack was then subject to non-uniform refinement to generate the final map.

For the wild-type TAP with ATP at 37°C, a combined Topaz model for both the inward-facing and outward-facing conformations was used to pick all the micrographs and subject to iterative 2D classification. *Ab initio* reconstruction with a maximum resolution set to 9Å yielded two distinct classes representing the outward- and inward-facing classes. Each conformation was separately subject to iterative rounds of heterogeneous refinement using the best reconstruction and a lower-resolution decoy reconstruction as input model. Non-uniform refinement of the best class for each conformation was then subject to 3D classification in Relion without alignment using a mask excluding the micelle. For the outward-facing state, the best class was selected based on the completeness of the TMDs. For the inward-facing state, the best stack was selected based on the completeness of the NBDs. The final particle stacks for both conformations were then subject to Bayesian particle polishing and then imported into cryoSPARC for local refinement to generate the final map.

FSC curves were generated in cryoSPARC and resolutions were reported based on the 0.143 criterion. Masking and B-factor sharpening were determined automatically in cryoSPARC during refinement.

#### Model building and refinement

The sharpened and unsharpened maps from local refinement were used for model building. Molecular models of TAP under active turnover conditions and of TAP(EQ) with ATP in the inward-facing wide conformation were initially built based on the cryo-EM structure of apo-TAP (PDB: 8T46). The molecular model of TAP with ATP and the b27 peptide was initially built based on the cryo-EM structure of TAP bound to the b27 peptide (PDB: 8T4F). The molecular model of TAP(EQ) with TAP in the outward-facing conformation was initially built based on the cryo-EM structure of TAP bound to the b27 peptide. The initial model was broken into four parts consisting of TAP1 TMD, NBD1, TAP2 TMD, and NBD2. Each model was rigid body fit into the density using ChimeraX^59^ and manually adjusted in Coot.^63^ The models were then iteratively edited and refined in Coot, ISOLDE,^60^ and PHENIX.^61^ The quality of the final models were evaluated by MolProbity.^65^ Refinement statistics are summarized in Table S1.

#### Figure preparation

Cryo-EM maps and atomic models generated using UCSF ChimeraX.^59^ Maps colored by local resolution were generated using cryoSPARC. Structural biology software used in this project was managed by SBGrid.^56^ All figures were prepared using Adobe Illustrator.

## Supplementary Material

MMC2

MMC1

Supplemental information can be found online at https://doi.org/10.1016/j.immuni.2025.08.003.

## Figures and Tables

**Table:** KEY RESOURCES TABLE

REAGENT or RESOURCE	SOURCE	IDENTIFIER

Chemicals, peptides, and recombinant proteins		

Fetal bovine serum, heat inactivated	GIBCO	CAT# 16–140-071
Antibiotic-antimycotic	GIBCO	CAT# 15240112
Sodium butyrate	Thermo Scientific	CAT# A1107936
Sf-900 II SFM medium	GIBCO	CAT# 10902088
Cellfectin II reagents	Invitrogen	CAT# 10362100
Freestyle 293 medium	GIBCO	CAT# 12338018
Lipofectamine 3000	Invitrogen	CAT# L3000008
GDN	Anatrace	CAT# GDN101
b27 peptide (RRYKSTEL)	Genscript	N/A
ATP	Sigma-Aldrich	CAT# P2645

Deposited data		

Raw micrographs of TAP bound to b27 and ATP	This paper	EMPIAR: 12685
Raw micrographs of TAP(EQ) bound to ATP	This paper	EMPIAR: 12686
Raw micrographs of TAP bound to ATP and ADP	This paper	EMPIAR: 12687
Cryo-EM map of TAP bound to b27	Lee et al. ^27^	EMDB: EMD-41029
Cryo-EM map of TAP bound to b27 and ATP in the inward-facing state	This paper	EMDB: EMD-49045
Cryo-EM map of TAP(EQ) bound to ATP in the inward-facing state	This paper	EMDB: EMD-49046
Cryo-EM map of TAP(EQ) bound to ATP in the outward-facing open state	This paper	EMDB: EMD-49047
Cryo-EM map of TAP(EQ) bound to ATP in outward-facing kinked state	This paper	EMDB: EMD-49048
Cryo-EM map of TAP bound to ATP and ADP in the inward-facing state	This paper	EMDB: EMD-49049
Cryo-EM map of TAP bound to ATP and ADP in the outward-facing open state	This paper	EMDB: EMD-49050
Coordinates of TAP bound to b27	Lee et al.^27^	PDB 8T4F
Coordinates of TAP bound to b27 and ATP in the inward-facing state	This paper	PDB 9N61
Coordinates of TAP(EQ) bound to ATP in the inward-facing state	This paper	PDB 9N62
Coordinates of TAP(EQ) bound to ATP in the outward-facing open state	This paper	PDB 9N63
Coordinates of TAP(EQ) bound to ATP in outward-facing kinked state	This paper	PDB 9N64
Coordinates of TAP bound to ATP and ADP in the inward-facing state	This paper	PDB 9N65
Coordinates of TAP bound to ATP and ADP in the outward-facing open state	This paper	PDB 9N66

Experimental models: Cell lines		

Sf9	ATCC	CRL-1711
HEK293S GnTI-	ATCC	CRL-3022

Recombinant DNA		

pEG TAP1-GFP/TAP2	Lee et al. ^27^	N/A
pEG TAP1-GFP/TAP2(EQ)	This paper	N/A

Software and algorithms		

SerialEM	Mastronarde et al.^52^	http://bio3d.colorado.edu/SerialEM
MotionCor2	Zheng et al.^53^	https://emcore.ucsf.edu/ucsf-software
CTFFIND4	Rohou et al.^54^	https://grigoriefflab.umassmed.edu/ctffind4
crYOLO	Wagner et al.^55^	https://cryolo.readthedocs.io/en/stable/index.html
Relion	Scheres et al.^56^	http://www2.mrc-lmb.cam.ac.uk/relion
cryoSPARC	Punjani et al.^57^	https://cryosparc.com
Csparc2star.py	Asarnow et al.^58^	https://doi.org/10.5281/zenodo.3576630.
Coot	Emsley et al.^59^	https://www2.mrc-lmb.cam.ac.uk/personal/pemsley/coot/
Phenix	Liebschner et al.^60^	https://www.phenix-online.org
Molprobity	Chen et al.^61^	http://molprobity.biochem.duke.edu
ChimeraX	Petterson et al.^62^	https://www.cgl.ucsf.edu/chimerax/
ISOLDE	Croll et al.^63^	https://tristanic.github.io/isolde/

Other		

CNBR-activated sepharose beads	GE Healthcare	17–0430-01
Glutathione Sepharose 4B	Fisher	45–002-065
Superose 6 Increase, 10/300 GL	Cytiva	29091596
R0.6/1 400 mesh Au holey carbon grids	Quantifoil	Q530AR-06

## Highlights

- Cryo-EM structures of human TAP reveal the ATP-dependent antigen transport mechanism
- Conformational changes in the outward-facing state facilitate peptide release
- Asymmetric opening of the NBD dimer upon ATP hydrolysis and phosphate release
