# Supplementary material for: Nucleotide-dependent conformational changes direct peptide export by the transporter associated with antigen processing
Source: Immunity. Author manuscript; Available in PMC 2025 Sep 11. (PMC12422388; doi:10.1016/j.immuni.2025.08.003)
Supplement: MMC1 [file NIHMS2104708-supplement-MMC1.pdf]

Immunity, Volume *58*

Supplemental information

Nucleotide-dependent conformational changes direct peptide export by the transporter associated with antigen processing

James Lee, Victor Manon, and Jue Chen

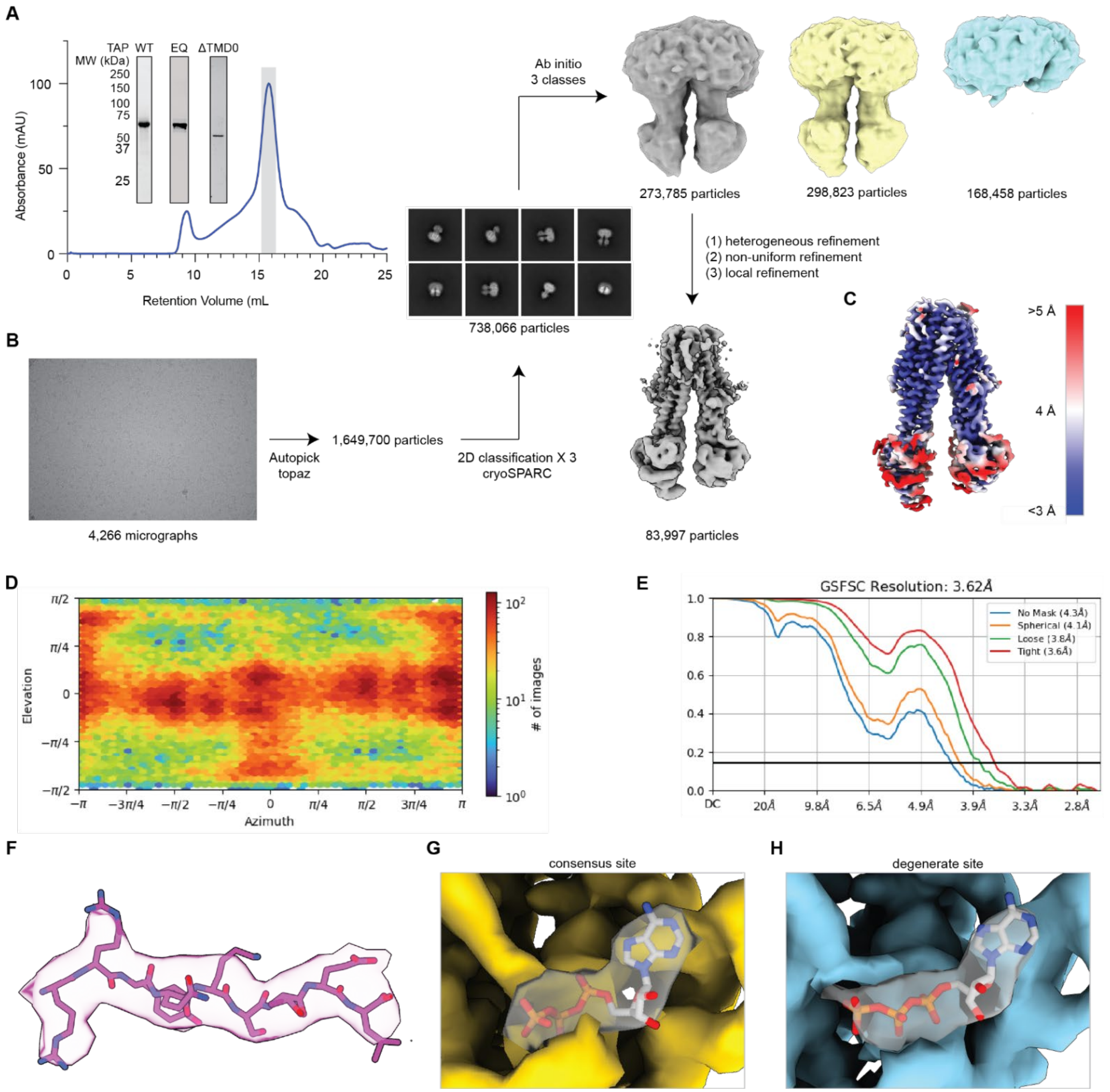

Figure S1: cryo-EM processing of wild-type TAP in the presence of ATP and the 9-mer peptide RRYQKSTEL at 4ºC, related to Figure 1.

(A) Gel filtration profile of WT TAP. Fractions taken for structural analysis are highlighted. SDS-PAGE of protein samples stained by Coomassie blue of WT TAP, TAP(EQ) and TAP ΔTMD0, which lacks both TMD0 domains (TMD0). **(B)** Summary of image processing. **(C)** Local resolution estimation. **(D)** Angular distribution of particles. **(E)** Fourier shell correlation curve. **(F-H)** Unsharpened cryo-EM density around the peptide (E) and nucleotide binding sites (F-G) contoured to 0.35 and 0.5, respectively.

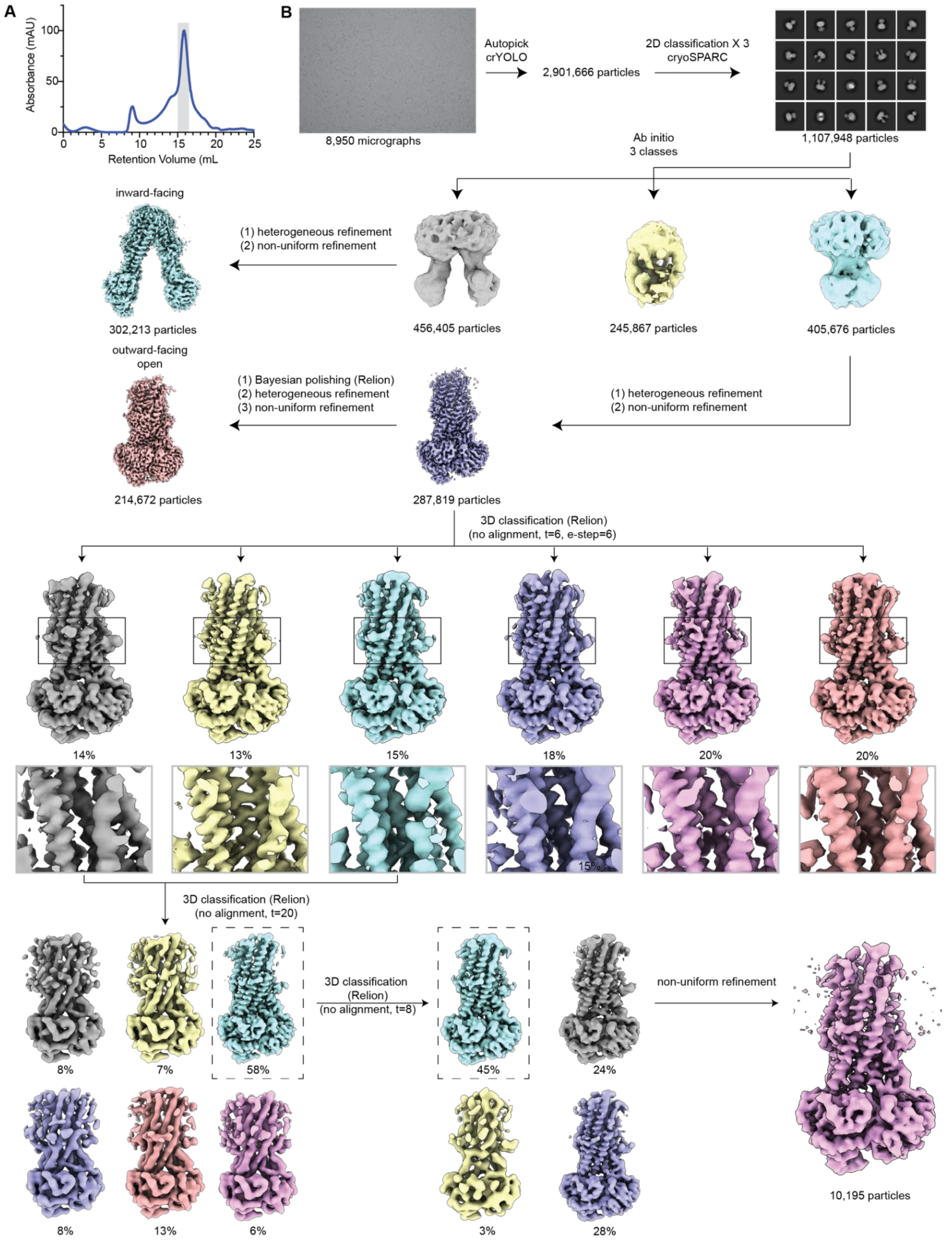

Figure S2: cryo-EM processing of TAP (EQ) bound to ATP, related to Figures 2 and 3.
(A) Gel filtration profile of TAP(EQ). Fractions taken for structural analysis are highlighted. **(B)** Summary of image processing.

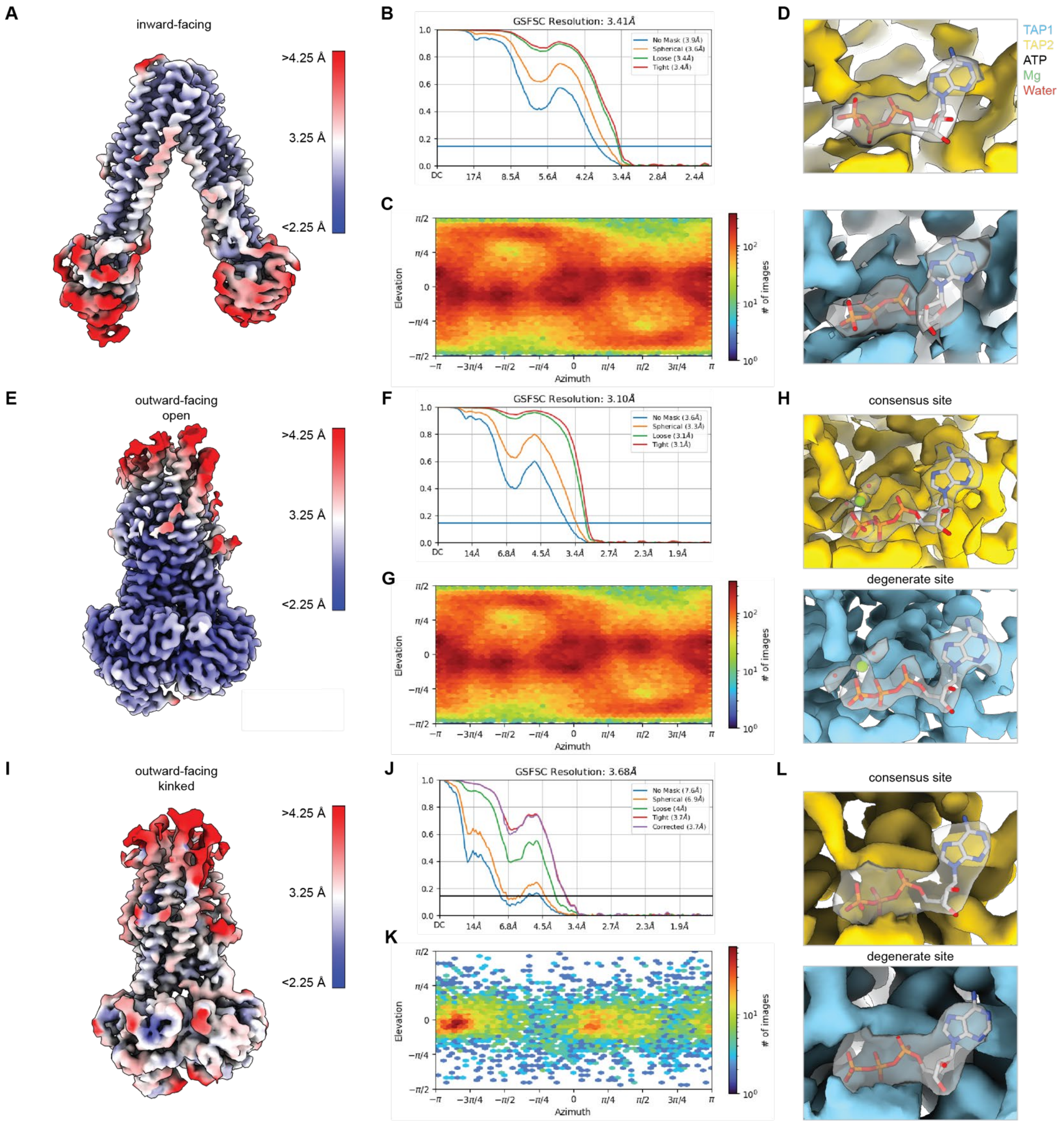

Figure S3: Cryo-EM analysis of TAP(EQ) in the presence of ATP, related to Figures 2 and 3.
(A-D) Local resolution estimation **(A)**, Fourier shell correlation curve **(B)**, angular distribution of particles **(C)**, and nucleotide density **(D)** of inward-facing TAP bound to ATP. Reconstructions are contoured to 0.4 SDs. **(E-H)** Local resolution estimation **(E)**, Fourier shell correlation curve **(F)**, angular distribution of particles **(G)**, and nucleotide density **(H)** of outward-facing TAP bound to ATP. Reconstructions are contoured to 0.6 SDs. **(I-L)** Local resolution estimation **(I)**, Fourier shell correlation curve **(J)**, angular distribution of particles **(K)**, and nucleotide density **(L)** of outward-facing TAP bound to ATP. Reconstructions are contoured to 0.4 SDs.

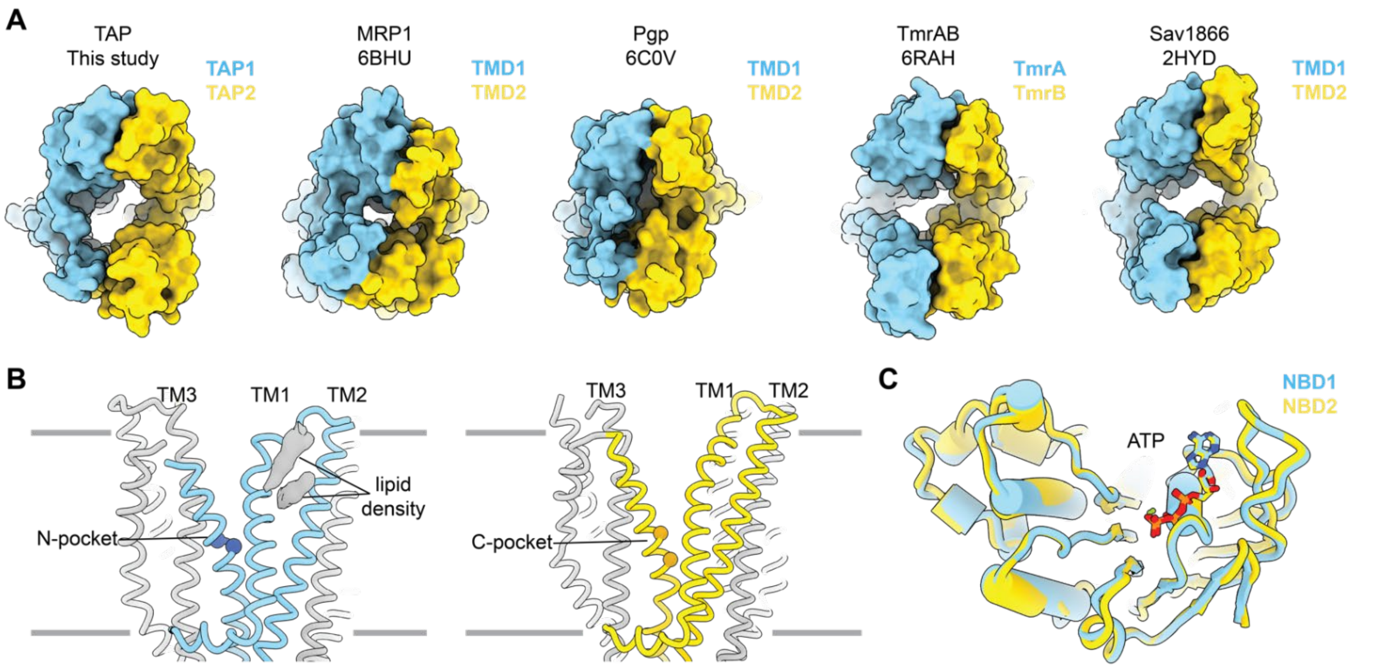

Figure S4: Structural features of outward-facing TAP(EQ), related to Figures 2 and 3.
(A) Comparison of outward-facing open TAP to other ABC transporters. Surface representation of various ABC transporters in the outward-facing state as viewed from the ER lumen in the case of TAP or from the extracellular space in the case of the others. Each half of the transporter is colored in sky blue or gold. **(B)** Zoom-in view of the lateral gates of the TAP transmembrane domains from the membrane. TAP1 (left) and TAP2 (right) TM1-3 are colored in sky blue and gold respectively. The alpha carbons corresponding to TAP1 W308 and Y309 in the N-pocket and TAP2 N269k and R273 in the C-pocket are shown as spheres. Density corresponding to putative lipids within the TAP1 lateral gate are marked. Reconstructions are contoured to 0.1. (C) Superposition of NBD1 and NBD2 of TAP(EQ).

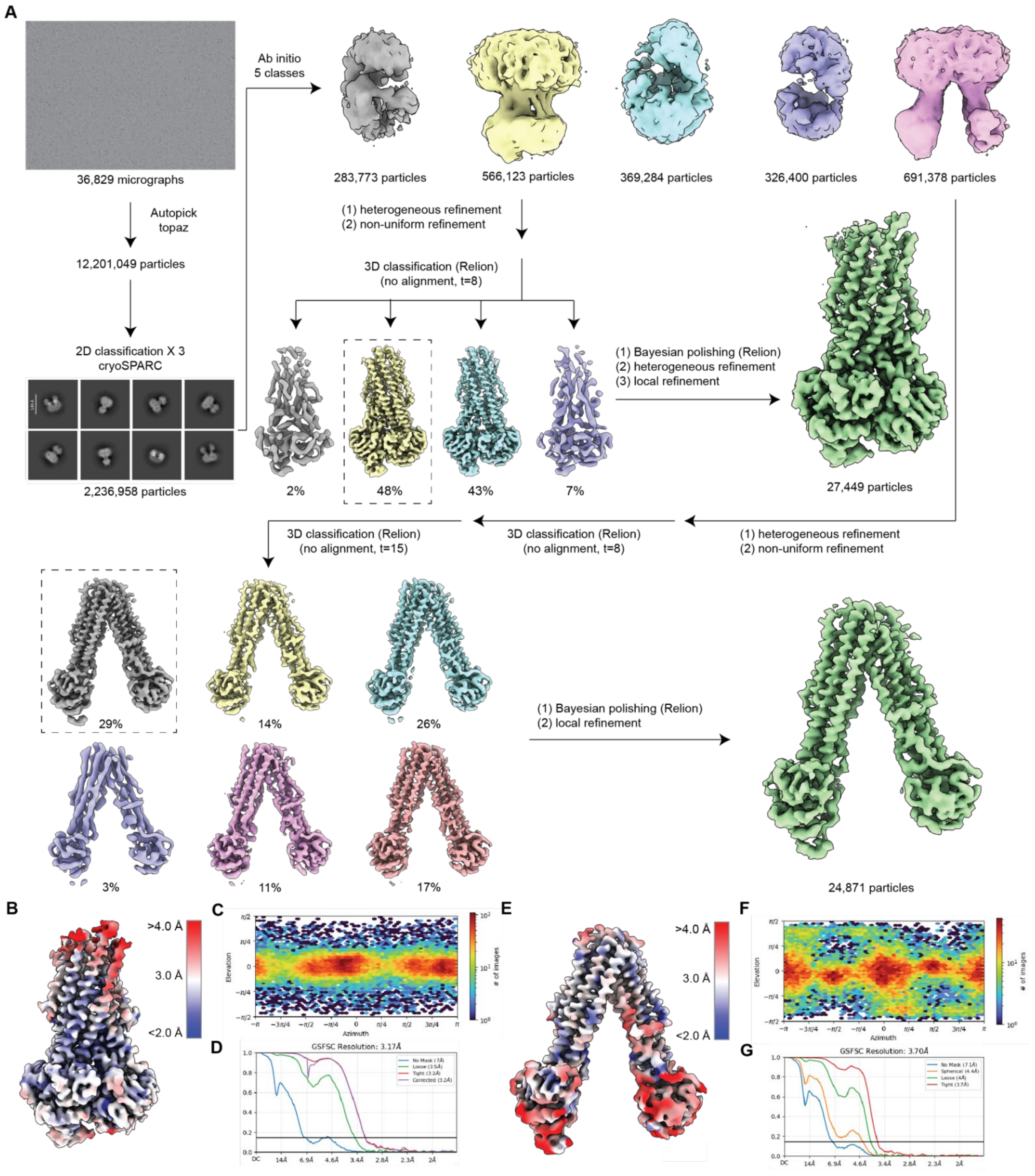

Figure S5: Cryo-EM analysis of wild-type TAP in the presence of ATP at 37ºC, related to Figures 4 and 5.
(A) Summary of image processing. **(B-D)** Local resolution estimation **(B)**, Fourier shell correlation curve **(C)**, and angular distribution of particles **(D)** of post-hydrolytic outward-facing TAP bound to ATP/ADP. Reconstructions are contoured to 0.13 SDs. **(E-G)** Local resolution estimation **(E)**, Fourier shell correlation curve **(F)**, and angular distribution of particles **(G)** of post-hydrolytic inward-facing TAP bound to ATP/ADP. Reconstructions are contoured to 0.14 SDs.

Table S1. Cryo-EM data collection, refinement, and validation statistics, related to STAR Methods.

	TAP + ATP + B27 peptide<br>Inward-facing<br><br>EMD-49045<br>PDB 9N61	TAP(EQ) + ATP<br><br>Inward-facing<br><br>EMD-49046<br>PDB 9N62	TAP(EQ) + ATP<br><br>Outward-facing<br><br>EMD-49047<br>PDB 9N63	TAP(EQ) + ATP<br><br>Outward-facing<br>Kinked<br>EMD-49048<br>PDB 9N64
**Data collection and processing**				
Magnification	81,000	105,000	105,000	105,000
Voltage (kV)	300	300	300	300
Electron exposure (e^{-}/Å^2)	50	50	50	50
Defocus range (μm)	0.8 to 1.5	0.8 to 2.0	0.8 to 2.0	0.8 to 1.5
Pixel size (Å)	1.09	0.847	0.847	0.847
Symmetry imposed	C1	C1	C1	C1
Initial particle images (no.)	1,649,700	2,900,666	2,900,666	2,900,666
Final particle images (no.)	83,997	302,213	214,672	10,195
Map resolution (Å)	3.6	3.4	3.1	3.7
FSC threshold	0.143	0.143	0.143	0.143
**Refinement**				
Initial model used (PDB code)	8T4F	8T46	9N62	9N63
Model resolution (Å)	3.8	3.5	3.1	3.9
FSC threshold	0.5	0.5	0.5	0.5
Map sharpening *B* factor (Å^2)	111.5	104.8	80	68.7
Model composition				
Non-hydrogen atoms	8864	8778	8569	8513
Protein residues	1130	1121	1096	1088
Water	0	0	4	4
Ligands	2	2	4	4
B factors (Å^2)				
Protein	95.03	100.04	74.34	87.67
Ligand	139.77	129.69	44.81	76.16
Water		N/A	43.00	75.75
R.m.s. deviations				
Bond lengths (Å)	0.004	0.004	0.007	0.003
Bond angles (°)	0.748	0.633	1.144	0.811
Validation				
MolProbity score	1.48	1.43	1.47	1.38
Clashscore	5.11	5.22	4.36	4.97
Poor rotamers (%)	0.10	0.11	0.32	0.76
Ramachandran plot				
Favored (%)	96.71	97.14	96.22	97.40
Allowed (%)	3.29	2.86	3.78	2.60
Disallowed (%)	0.00	0.00	0.00	0.00

Table S1. Cryo-EM data collection, refinement, and validation statistics, related to STAR Methods.

	WT TAP + ATP<br><br>Inward-facing<br><br>EMD-49049<br>PDB 9N65	WT TAP + ATP<br><br>Outward-facing<br><br>EMD-49050<br>PDB 9N66
**Data collection and processing**		
Magnification	105,000	105,000
Voltage (kV)	300	300
Electron exposure (e–/Å^2)	50	50
Defocus range (μm)	0.8 to 2.0	0.8 to 2.0
Pixel size (Å)	0.86	0.86
Symmetry imposed	C1	C1
Initial particle images (no.)	5,485,574	5,485,574
Final particle images (no.)	24,871	27,449
Map resolution (Å)	3.7	3.2
FSC threshold	0.143	0.143
**Refinement**		
Initial model used (PDB code)	8T46	9N63
Model resolution (Å)	4.0	3.4
FSC threshold	0.5	0.5
Map sharpening *B* factor (Å^2)	93.2	53.0
Model composition		
Non-hydrogen atoms	8778	8532
Protein residues	1121	1092
Ligands	4	4
B factors (Å^2)		
Protein	49.99	57.16
Ligand	92.44	47.33
R.m.s. deviations		
Bond lengths (Å)	0.004	0.003ss
Bond angles (°)	0.877	0.720
Validation		
MolProbity score	1.40	1.41
Clashscore	3.74	3.91
Poor rotamers (%)	0.32	0.33
Ramachandran plot		
Favored (%)	96.42	96.49
Allowed (%)	3.58	3.51
Disallowed (%)	0.00	0.00
